# Noise Reduction for Single-Shot Grating-Based Phase-Contrast Imaging at an X-ray Backlighter

**DOI:** 10.3390/jimaging7090178

**Published:** 2021-09-05

**Authors:** Stephan Schreiner, Bernhard Akstaller, Lisa Dietrich, Pascal Meyer, Paul Neumayer, Max Schuster, Andreas Wolf, Bernhard Zielbauer, Veronika Ludwig, Thilo Michel, Gisela Anton, Stefan Funk

**Affiliations:** 1 Erlangen Centre for Astroparticle Physics (ECAP), Friedrich-Alexander Universität Erlangen-Nürnberg, Erwin-Rommel-Straße 1, 91058 Erlangen, Germany; bernhard.akstaller@fau.de (B.A.); lisa.dietrich@fau.de (L.D.); max.schuster@fau.de (M.S.); andreas.wolf@fau.de (A.W.); veronika.ludwig@fau.de (V.L.); thilo.michel@fau.de (T.M.); gisela.anton@physik.uni-erlangen.de (G.A.); s.funk@fau.de (S.F.); 2 Karlsruhe Institute of Technology, Institute of Microstructure Technology, Hermann-von-Helmholtz-Platz 1, 76344 Eggenstein-Leopoldshafen, Germany; pascal.meyer@kit.edu; 3 GSI Helmholtzzentrum für Schwerionenforschung GmbH, Planckstraße 1, 64291 Darmstadt, Germany; P.Neumayer@gsi.de (P.N.); B.Zielbauer@gsi.de (B.Z.)

**Keywords:** grating-based phase-contrast, single-shot X-ray phase-contrast imaging, X-ray backlighter, X-ray generators and sources, image processing, image quality improvement

## Abstract

X-ray backlighters allow the capture of sharp images of fast dynamic processes due to extremely short exposure times. Moiré imaging enables simultaneously measuring the absorption and differential phase-contrast (DPC) of these processes. Acquiring images with one single shot limits the X-ray photon flux, which can result in noisy images. Increasing the photon statistics by repeating the experiment to gain the same image is not possible if the investigated processes are dynamic and chaotic. Furthermore, to reconstruct the DPC and transmission image, an additional measurement captured in absence of the object is required. For these reference measurements, shot-to-shot fluctuations in X-ray spectra and a source position complicate the averaging of several reference images for noise reduction. Here, two approaches of processing multiple reference images in combination with one single object image are evaluated regarding the image quality. We found that with only five reference images, the contrast-to-noise ratio can be improved by approximately 13% in the DPC image. This promises improvements for short-exposure single-shot acquisitions of rapid processes, such as laser-produced plasma shock-waves in high-energy density experiments at backlighter X-ray sources such as the PHELIX high-power laser facility.

## 1. Introduction

Phase-contrast imaging allows extending conventional attenuation imaging with information about the refractive properties of an object. There is a wide variety of X-ray phase-contrast imaging techniques available. These include crystal interferometers [1,2], edge-illumination [3,4], propagation-based imaging [5,6,7,8,9,10] and grating-based techniques [11,12,13,14]. Grating-based techniques were initially developed in the research field of medical imaging [14,15] and non-destructive testing [16,17,18]. To date, these techniques have also been used in the field of laboratory astrophysics with the long-term aim of imaging processes on extremely short time scales. Such a process is, e.g., a laser-driven plasma shock wave in a high-energy density experiment. Using a laser-driven X-ray backlighter source for probing these objects enables one to acquire sharp images of these ultra-fast processes. If for such an object the X-ray absorption is low, imaging could benefit from the single-shot phase-contrast technique, since it is able to enhance contrast by probing the phase-shifting properties of a plasma [19,20]. Experiments striving the goal of imaging laser-driven plasma shock waves with phase-contrast were performed on high-power laser facilities such as Omega EP [21] and CELIA [22] with a Talbot–Lau setup consisting of three gratings. At the PHELIX facility, the propagation-based phase-contrast imaging of laser-induced shock waves [23,24] was conducted. As grating-based phase-contrast imaging offers advantages compared to propagation-based techniques under certain conditions [25], such as, e.g., higher sensitivity to changes in the phase, test measurements at the PHELIX facility using gratings were performed and presented in [26]. There, static samples, imaged at high magnification using grating-based phase-contrast imaging, were evaluated.

Grating-based phase-contrast imaging requires a reference image, i.e., an image without object, taken with the same imaging system under similar conditions to those under which the object was measured. This is difficult to realize in the harsh environment of a backlighter experiment, where the X-ray spectrum and the source position changes from shot to shot. Stutman et al. [27] proposed the use of synthetic reference images. As a proof of principle measurement at a laboratory X-ray tube with a three-grating setup, they combined a single-shot object image with reference images acquired using phase-stepping. In 2020, Bouffetier et al. [22] managed to average a large amount of images taken with a three-grating Talbot–Lau setup at a high-repetition-rate backlighter K-*α* source and reconstruct low-noise images from that. If the X-ray source has sufficient spatial coherence, the source grating can be omitted [14,28], allowing us to use a two-grating setup. This increases the photon flux. The imaging setup is sensitive to the spectrum and the exact position of the X-ray source. The lack of reproducibility between the reference images makes it difficult to process them into one reconstruction.

The objective of this work is to compare two methods for improving image quality by using multiple reference images acquired with different X-ray spectra and source positions. The standard method combines multiple references into one average reference image before the reconstruction [22]. The other method combines the different images after reconstruction. The results show that, while the second approach provides slightly better contrast-to-noise ratios in the DPC image, both methods improve the image quality.

## 2. Materials and Methods

### 2.1. Grating-Based Phase-Contrast Imaging

The imaging system consists of an X-ray backlighter, driven by a shorted-pulsed laser with a pulse duration of 700 fs, two gratings mounted on a rigid setup, an object placed in front of the first grating and an imaging plate detector. A more detailed explanation of the experimental setup is given in Section 2.2.

Grating-based phase-contrast imaging is based on the analysis of self images of a periodic structure, in this case a line grating. The self image of the grating occurs at certain distances, called fractional Talbot distances, downstream from the grating [29,30]. A phase shift, induced by an object placed in the beam path, causes a shift in the self image. However, the pattern is usually smaller than the spatial resolution of the detector. To still be able to resolve the pattern, a second grating G2 is placed at a distance where the period of the pattern approximately matches the period of the G2 grating due to magnification. By overlaying two periodic structures with small deviations in the period or relative angle between the periodic structures, a Moiré pattern occurs [31]. The period of the Moiré pattern can be adjusted in a way that it is large enough to be resolvable with the detector. This pattern carries the same phase information as the Talbot pattern itself, yet with a lower resolution [32]. The contrast of these fringes is referred to as visibility *V*.

**Figure 1:**
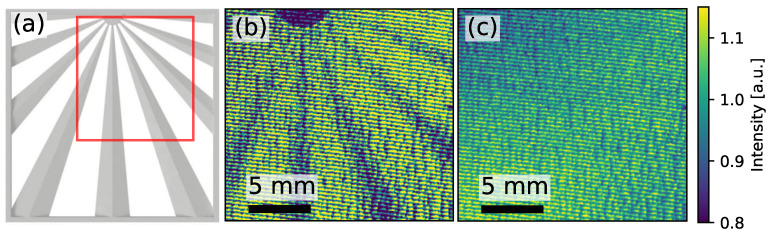
(**a**) 3D rendering of the used sample. The shading within one branch indicates an increasing, decreasing thickness, respectively. The frame and the semicircle on the upper frame have a constant thickness. Its overall dimension is 42.5 × 42.5 mm and was made of a polyacrylic mixture using stereolithography. The red frame marks the area which is presented in this work. The images (**b**,**c**) show a section of the object image and reference image number one. The Moiré stripes are tilted slightly clockwise from the horizontal axis. The scale indicates the size in the detector plane.

In Fourier space, the Moiré pattern can be separated from the object structures [33]. The separated information can be processed to three image modalities [32,34,35], the transmission image, the differential phase image and the darkfield image. To reconstruct these images, a reference image is required, i.e., an additional measurement with only the gratings in the beam path and no other object. Figure 1b,c shows an exemplary object and reference image, acquired at an X-ray backlighter source. The test object, see Figure 1a, consists of long double wedges arranged in a star-like fashion.

The three images are reconstructed from the object image $I_{\mathrm{obj}}\left(x_{i}\right)$ and the reference image $I_{\mathrm{ref}}\left(x_{i}\right)$, $x_{i}$ being the position of pixel *i*, by evaluating the Moiré signature Ixi=Ai1+Visin2πλm·xi+ϕi, with amplitude $A_{i}$, visibility $V_{i}$, phase $\phi_{i}$ and Moiré wavelength $\lambda_{m}$. The transmission image *T*, the differential phase image DPC and the darkfield image *D* are then defined as

$$\begin{array}{cc} T & =\frac{A_{i,\;\mathrm{obj}}}{A_{i,\;\mathrm{ref}}},\\ \mathrm{DPC} & =\phi_{i,\;\mathrm{obj}}-\phi_{i,\;\mathrm{ref}\;},\\ D & =\frac{V_{i,\;\mathrm{obj}}}{V_{i,\;\mathrm{ref}\;}}. \end{array}$$

The fast Fourier transform of object and reference images show three prominent peaks in frequency space. A central zeroth order peak, located around the zero and two first-order harmonic peaks, symmetrically located around the zeroth order at the spatial frequency of the Moiré pattern. The method of selecting the data in the respective peak area for the image reconstruction is conducted as introduced in [35]. For the transmission image *T*, the Moiré stripes are removed by deleting the first-order harmonic peaks, resulting in standard radiography. The differential phase-contrast image DPC stems from the phase shifting properties of the object [13,36] and is calculated using the first-order harmonics peaks. The dark-field image *D* indicates, among other things, the small angle scattering of the sample [17,37] and is calculated by processing the zeroth and first-order harmonics peaks. Since the investigated object is made of a homogeneous material which shows no dark-field signal, this modality will not be discussed any further.

**Figure 2:**
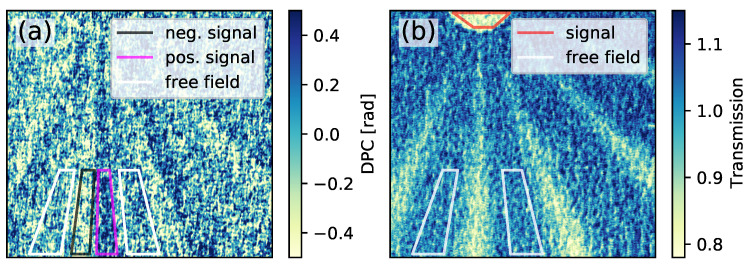
Reconstructed DPC (**a**) and transmission image (**b**) using the reference image one. Although the Moiré stripes are completely removable, the image is still noisy due to the low photon flux. The colored boxes mark the regions of interest (ROI) which are used for the evaluation process of the image.

Figure 2 shows a reconstructed DPC (a) and transmission image (b). The corresponding object and reference images are presented in Figure 1b,c. As one single double wedge of the object consists of a rising and a falling side, the differential phase-contrast image has a uniform negative signal on one side, and a uniform positive signal on the other side. The marked regions of interest (ROI) will later be used for the image quality evaluation. In the transmission image, the signal of the double wedge is symmetric between the two sides of the double wedge. However, since the wedges become smaller and flatter towards the center of the star-like shape, the absorption contrast decreases, as can be seen in Figure 2b.

### 2.2. Experimental Setup

For the measurements, a tungsten wire of 5 μm diameter was used as a backlighter source. The laser system used to drive the backlighter was the Petawatt High-Energy Laser for Heavy Ion Experiments (PHELIX) [38] at the GSI Helmholtzzentrum für Schwerionenforschung GmbH. This laser system delivers a 700 fs pulse with energies between approximately 25 J and 30 J. The backlighter delivers a very short X-ray flash with sufficient intensity for imaging. The spectrum of this backlighter setup at PHELIX was analyzed by Borm [39]. The dominating X-ray energy at PHELIX was determined in [40] using propagation-based phase-contrast imaging. In [26], a grating-based phase-contrast imaging setup, optimized for the dominating energy range, was introduced, evaluated, and the dominating energy verified. Accordingly, we expect an effective energy of 11 keV with a fluctuation of approximately 1 keV. In addition to the spectrum, the source position is fluctuating as well. The magnitude of this lateral fluctuation is given in [26] with 20 μm.

For the X-ray detection, Fuji BAS type SR imaging plates with a spatial resolution of 109 μm [41] were mounted in a box, shielded from visible light with a 8 μm-thick foil fabricated from high-purity aluminum. For the digitization process, a scanner with a 50 μm pixel pitch was used, which is far below the spatial resolution of the imaging plates. The gray-scale values of the scanned imaging plates were converted to photostimulated luminescence (PSL) units, as proposed in [42].

**Figure 3:**
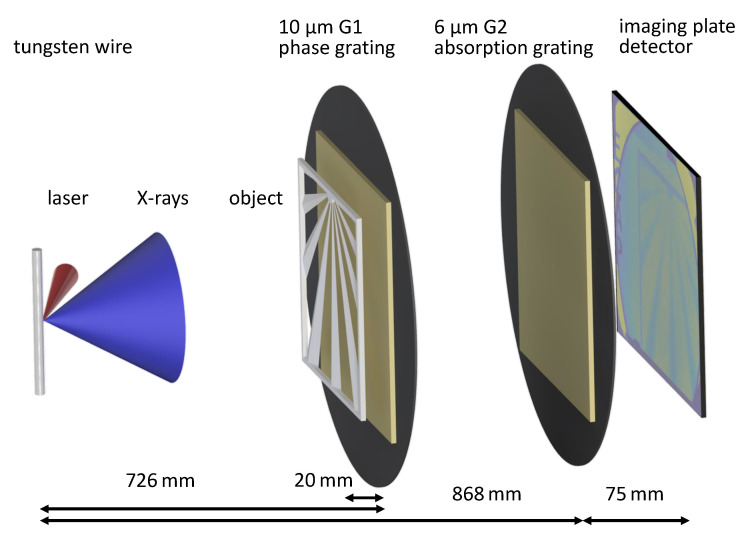
3D rendering of the imaging setup (not to scale). The 5 μm-thick tungsten backlighter wire, that serves as a broadband X-ray source, is driven by a 700 fs infrared laser pulse from the PHELIX high-power laser system. Both gratings are mounted on the portable (yet rigid) setup at distances of 726 mm and 868 mm from the source. The object is placed at approximately 20 mm in front of G1. The detector is mounted 75 mm behind the G2 grating. The setup is designed to yield a Moiré fringe period of 350 μm in the detector plane, with a visibility of the Moiré pattern of up to 16%.

A schematic of the Moiré imaging setup is shown in Figure 3. A similar setup was also used in [26], to image a 25 μm-thick polyimide foil with a magnification of approximately 47. The used gratings were manufactured by the Institute of Micro Structure Technology (IMT) at the Karlsruhe Institute of Technology (KIT) using the deep X-ray LIGA process [43,44]. The G1 grating with a period of 10 μm was manufactured as a phase grating with 25 μm high SU-8 lamellae onto a 500 μm-thick polyimide wafer. The G2 grating with a period of 6 μm was manufactured as an absorption grating with gold lamellae of a height of 150 μm onto a 500 μm-thick graphite wafer. The discontinuous structure of the Moiré stripes, as can be seen in Figure 1, is caused by imperfections of the G2 grating and/or by 2 μm-wide resist bridges which are necessary to stabilize the thick gold lamellae.

The setup was designed for an X-ray energy of approximately 11 keV, which roughly corresponds to the dominating X-ray energy [40]. The G1 grating for this energy is approximately π-shifting, resulting in a Talbot pattern with twice the frequency of the grating. The distances were calculated so that the G1 Talbot pattern, magnified in the G2 plane, has approximately the same period in the G2 grating. This assures that the required Moiré period can be adjusted [31]. Furthermore, the distances between the two gratings match the Talbot distance of the G1 grating, calculated for the expected 11 keV. The alignment process of the portable interferometer with respect to the backligher source was performed using the fast-alignment method described in [28].

The presented dataset consists of six images—one image with a sample and five without. Hence, five different reference images are available for one object measurement. For each measurement, the tungsten backlighter wire was replaced. Due to the cool-down time of the laser system, the ventilation and later on the evacuation of the target chamber, where the backlighter wire and the imaging setup were placed, a shot rate of five to six shots per day was feasible. The laser spot shape and its position were kept as constant as possible but was not perfectly reproducible. This results in varying image quality. Some measurements suffer from blurring, such that they cannot be used for image reconstruction. The mean intensity of the used images varies by a factor of up to 3.7 in the free field. As the photon flux is naturally restricted at these X-ray sources, the images exhibit noise. The mean visibility of the Moiré pattern in the object and reference images ranges between 6% and 16% with a standard deviation of 3%. As higher visibility correlates with a lower noise level in the DPC image [45], the reconstructed images will differ regarding their noise. In total, this shows that the backlighter source poses challenges for the experimental design and image analysis.

For the image reconstruction, all reference images were matched onto the object image via three markers around the G2 grating. These markers are required and hence fabricated during the grating manufacturing process. They have a size of a few hundred micrometers and are cross or tip-like. These markers are matched with subpixel accuracy using a cross-correlation algorithm. Subsequently, each reference image is translated, rotated, and scaled until it matches the object image. To reduce salt-and-pepper noise, a move-mean algorithm with a 2×2 binning was applied. Similar to [17], a row-by-row offset correction was applied by fitting a first-order polynomial, onto the free field to obtain a constant background in the reconstructed DPC images. As the photon flux varies slowly across the field of view, the offset correction for the transmission image is performed by fitting a two-dimensional second-order polynomial onto the free field. This way, the gradient in the background of the measurement data, which is clearly visible in Figure 1c, was completely corrected in the reconstructed transmission image, as can be seen in Figure 2b. Both image modalities have a noisy appearance due to the limited flux of X-ray photons from the backlighter source.

### 2.3. Image Evaluation

In laboratory measurements with conventional X-ray tubes, multiple images can be acquired within a few seconds. By averaging over all acquired images, the photon statistic can be increased. This method requires a rigid setup where none of the components move during the measurement process. Hence, in the experiments performed at X-ray backlighter facilities, where the X-ray spectra, Moiré visibility and position of the source point may change from shot to shot, this is not readily possible. Thus, two different approaches of averaging data, acquired at the PHELIX facility, are investigated herein. One approach follows the just described method of averaging the acquired reference images before the reconstruction is performed. This method will subsequently be referred to as average-then-reconstruct (ATR). As an alternative approach, the reconstruction is performed for each single reference image and then the resulting images are averaged. This will be referred to as reconstruct-then-average (RTA). With five reference images available, there are 31 different possibilities to combine the reference images or reconstructed images.

Since the reference images differ in their mean intensity and Moiré visibility, the weighted arithmetic mean of the reference images or reconstructed images was used, respectively. The weighed arithmetic mean is given by

$$\bar{y}=\sum_{i}\frac{w_{i}^{T,\mathrm{DPC}}}{\sum_{j}w_{j}^{T,\mathrm{DPC}}}\cdot y_{i}\;,$$

with $y_{i}$ being the *i*-th reference image normalized with respect to the intensity (ATR method) or the *i*-th reconstructed image (RTA method), $w_{i}^{T,\mathrm{DPC}}$ and $w_{j}^{T,\mathrm{DPC}}$ an image modality dependent weight factor and $\bar{y}$ the averaged reference or reconstruction. The indices *i* and *j* are chosen according to the regarded reference image combination. The weight factor $w_{i}^{T,\mathrm{DPC}}$ and $w_{j}^{T,\mathrm{DPC}}$ are chosen for both image modalities differently, as the noise in the different image modalities depends on different parameters. The noise in the transmission image *T* depends on the photon flux. Hence, the weighting factor $w_{i}^{T}$ was calculated using Poisson statistics to:

$$w_{i}^{T}=\mu_{i,\;free\;field}^{\mathrm{wafer}}\;,$$

with $\mu_{i,\;free\;field}^{\mathrm{wafer}}$, the mean intensity value of a chosen ROI outside the Moiré fringe pattern of the *i*-th reference image. The noise in the DPC image additionally relies on the visibility of the Moiré pattern [45]. Accordingly, the weighting factor $w_{i}^{\mathrm{DPC}}$ is defined as

$$w_{i}^{\mathrm{DPC}}=\mu_{i,\;free\;field}^{\mathrm{wafer}}\cdot \mu_{i,\;V},$$

with $\mu_{i,\;V}$ the mean visibility of the *i*-th reference image within the area of image reconstruction.

To evaluate the impact of the RTA and ATR methods on the final images three image parameters are evaluated for every reconstructed image: the image noise, the contrast and the contrast-to-noise ratio (CNR). The noise is quantified by the standard deviation (STD) of the intensity in a free field ROI $\sigma_{\mathrm{free}\;\mathrm{field}}$. Subsequently, the noise is referred to as STD. The contrast is defined as

$$\mathrm{contrast}=|\mu_{\mathrm{signal}}-\mu_{\mathrm{free}\;\mathrm{field}}|,$$

with $\mu_{\mathrm{signal}}$ and $\mu_{\mathrm{free}\;\mathrm{field}}$ being the mean value of a signal ROI and a free field ROI, respectively. The CNR is defined as

$$\mathrm{CNR}=\frac{|\mu_{\mathrm{signal}}-\mu_{\mathrm{free}\;\mathrm{field}}|}{\sigma_{\mathrm{free}\;\mathrm{field}}}.$$

The ROIs used to calculate the values are shown in Figure 2. To be able to assess the CNR in more detail, the contrast and the standard deviation are separately evaluated for the selected free field.

## 3. Results

The standard deviation, contrast and contrast-to-noise ratio were initially investigated for the positive DPC signal. As the used object also provides a negative DPC signal, the findings were verified by calculating the CNR of the corresponding ROI. The transmission image is analyzed in a similar way. The ROIs for this evaluation process are marked in Figure 2.

**Figure 4:**
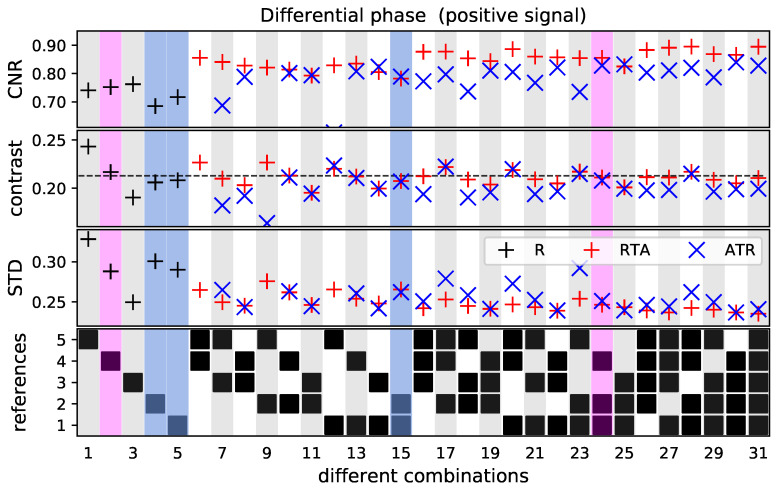
CNR, contrast and STD for the ROI of the positive DPC signal, marked in Figure 2a. The STD and contrast values are given in the radian. The plot in the last row indicates which reference images were used for deriving the values in the respective column. The red and blue markers show the results for reconstruct-then-average (RTA) and average-then-reconstruct (ATR), respectively. The black markers indicate the values for which only one reference image is used. The black dashed line indicates the mean contrast of the first five combinations. For the combinations 6, 9 and 12, the ATR method results in images with high standard deviation and low CNR, which are not within the range of the axis. Furthermore, the contrast is for the combinations 6 and 9 not within the range of the axis. The values in the colored columns were used for a detailed investigation.

The values of the positive signal and the free field in the differential phase-contrast image are shown in Figure 4. The Figure is divided into four vertically arranged subplots with 31 columns each. In the lowest subplot, the combinations of the five reference recordings are specified. Here, the y axis marks the different reference images, and the 31 combinations are given along the x axis. The upper plots present the CNR, contrast and STD values. The black markers show the calculated values, if only one reference image is used for reconstruction. The red and blue markers show the RTA and ATR method, respectively.

It can be seen that the STD with an amplitude of approximately 0.27 rad is of the same order of magnitude as the contrast. Hence, the CNR ranges in a low regime of approximately 0.8. Nevertheless, it can be noted, that for both averaging methods the STD decreases and the CNR increases by using more reference images. The retrieved contrast converges for both methods to the black dashed line, which indicate the mean value of the first five combinations. For some combinations, where the fifth reference image is used (combination 6, 7, 9 and 12), the ATR method results in images with a significantly high standard deviation and low CNR compared to the RTA images. Generally, the CNR values derived with the RTA method are for nearly all combinations higher than the ATR method. Excluding the just mentioned combinations 6, 7, 9 and 12, the CNR is for the RTA method on average approximately 7% higher compared to the ATR method.

For a detailed evaluation of the improvement of the STD and CNR values, three reference images were selected (marked in blue and magenta). The single reference images were chosen in such a way that, if they were used on their own for the reconstruction, the STD, contrast and CNR values would be nearly the same. First, the average of the blue-marked combinations four and five is compared with combination 15. If these two references are combined, the STD decreased for both reconstruction methods by approximately (10 ± 2)%. As the derived contrast stays nearly the same, the CNR improves by approximately (12 ± 2)%. By additionally considering the magenta-marked reference image four, the STD decreases by (16 ± 1)% and the CNR increases by (19 ± 3)% compared to the mean STD and CNR of combination two, four and five.

**Figure 5:**
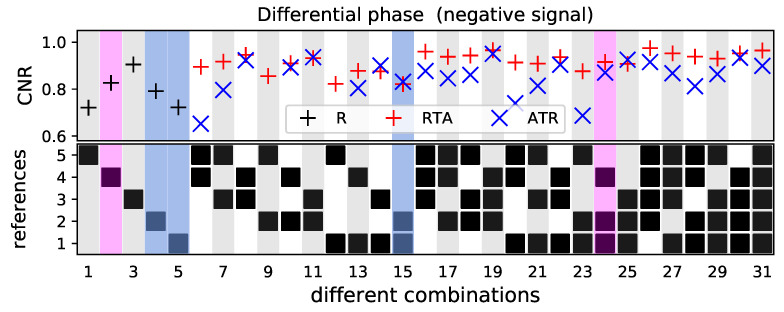
CNR for the ROI with the negative DPC signal, marked in Figure 2a. The STD and contrast values are given in radian. The plot in the last row indicates which reference images were used for deriving the values in the respective column. The black markers indicate the values if only one reference image is used. The red and blue markers show the results for the RTA and ATR method, respectively. For combinations 9 and 12, the ATR method results in images with high standard deviation and low contrast and CNR, which are not within the range of the axis. The values in the colored columns are used for a detailed investigation.

The observed tendency can be validated with the negative DPC signal, marked in black in Figure 2. The CNR investigated here is in the same range as the CNR for the positive signal (see Figure 5) and increases by using more reference images. Similarly, the RTA method leads most combinations to higher CNR values. Similarly to the positive DPC signal, the combinations where the reference image five is included, the CNR is with the ATR method but significantly lower compared to RTA method. Excluding the same combinations 6, 7, 9 and 12, the CNR is for the RTA method on average approximately 7% higher compared to the ATR method.

By comparing the mean CNR of combinations four and five with combination 15, the value increases by (9 ± 5)%. By including the magenta-marked fourth reference image in the calculations, the CNR increases for the ATR method by approximately (11 ± 4)% and for the RTA method by approximately (17 ± 5)%.

**Figure 6:**
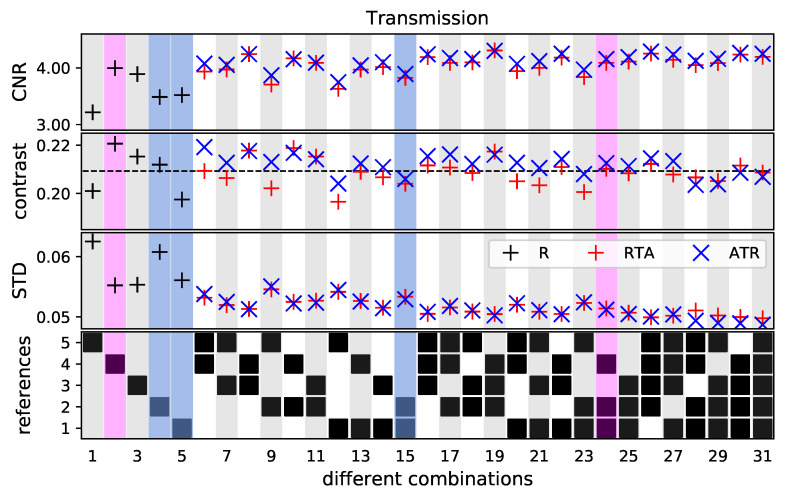
CNR, contrast and STD for the ROI of the transmission image, marked in Figure 2b. The STD and contrast values are given in values of relative transmission in percent. The plot in the last row indicates which reference images were used for deriving the values in the respective column. The red and blue markers show the results for the RTA and ATR, respectively. The black markers indicate the values if only one reference image is used. The black dashed line indicates the mean contrast of the first five combinations. The values in the colored columns are used for a detailed investigation.

For the reconstructed transmission images, the values can be seen in Figure 6. The presentation of the data is similar to the that of the positive DPC signal in Figure 4. The STD, contrast and CNR show also in this image modality the same overall behavior. The standard deviation decreases while the contrast evens out to the mean contrast of the first five combinations. The ATR method results for most combinations in identical or slightly higher contrast values. As the STD values are nearly identical for both methods, the slightly higher contrast leads to higher CNR values for the ATR method.

By taking a closer look at the same combinations investigated in the DPC image (reference one, two and four), it can be seen that the STD and contrast varies on a larger scale compared to the values retrieved in the DPC image for these images. The combined reference one and two (combination 15) has an approximately (9 ± 5)% lower STD compared to the mean STD of reference images one and two. The CNR increases by approximately (9 ± 1)%. Adding the magenta-marked reference image four, which has a higher initial CNR value due to the high contrast, the STD declines by (11 ± 4)% and the CNR improves by approximately (12 ± 5)%.

## 4. Discussion and Conclusions

The evaluation of the dataset shows that averaging reference images can significantly increase the contrast-to-noise ratio (CNR) in the reconstructed images taken at an X-ray backlighter source. For the DPC image, the reconstruct-then-average (RTA) method results in a higher CNR compared to the average-then-reconstruct (ATR) method. Excluding the outliers, which would make the discrepancy even greater, the difference in the CNR is on average approximately 7%. One possible explanation for this circumstance could be that averaging reference images with locally shifted Moiré pattern results in a pattern which is smeared out. Hence, the visibility of the Moiré pattern decreases [46] which causes increased noise in the DPC image [45]. Furthermore, the retrieved differential phase might suffer inaccuracies due to the altered shape of the reference pattern. For the CNR in the transmission image, both methods achieve nearly equal improvements. Here, the ATR method leads on average to an approximately 2% higher CNR compared to the RTA method.

**Figure 7:**
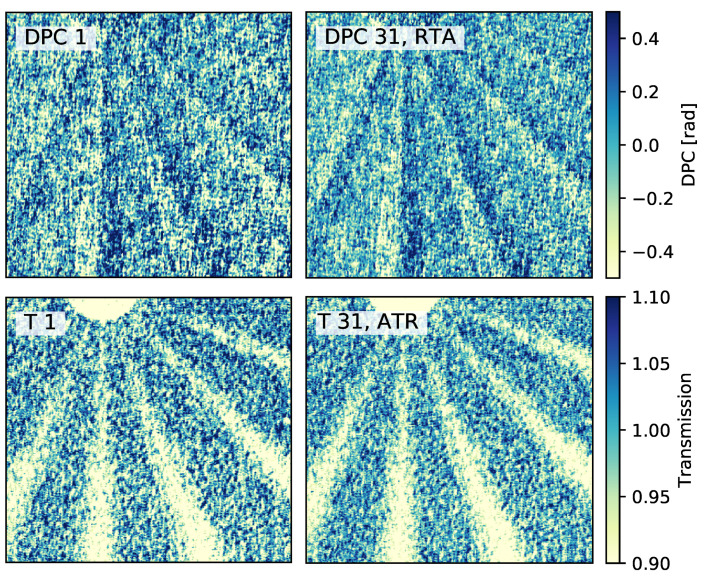
Two retrieved DPC images (DPC 1 and DPC 31, RTA) and transmission images (T 1 and T 31, ATR) for a visual comparison of the improved image quality. For the DPC image comparison, an image retrieved using reference image 5 is shown on the left (DPC 1) and on the right an image retrieved using all reference images and the RTA method (DPC 31, RTA). It can be seen with the naked eye that the noise is reduced and hence, the double wedges are easier to recognize. For the comparison of the transmission image, the image retrieved with reference image 5 (T 1) is again compared with the image retrieved using the ATR method and reference image combination 31 (T 31, ATR). Here, hardly any improvements are visible. The lower STD of the right image can vaguely be seen in the free field.

For a visual comparison of the derived image quality improvement, two DPC and two transmission images are presented in Figure 7. The images displayed on the left side are reconstructed using reference image five. On the right side, the shown images are chosen with regard to the lowest STD and highest SNR. The quality improvement of the DPC image can clearly be seen. Due to the lower STD, the double wedges are easier to recognize. The difference between the two transmission images can hardly be seen. There is only a vague improvement of the noise in the free field.

In both image modalities, the contrast seems to converge for more used reference images to the average contrast derived from the reconstructed images, which were generated using one reference image for the image retrieval.

Hence, if the contrast converges to a certain value and the standard deviation decreases with more the reference images used, the CNR might increase even further if more reference images were available. Nevertheless, this method is meant for imaging fast dynamic processes with Moiré imaging. The circumstance under which only one object image is available will limit the CNR at a certain point.

In conclusion, both presented methods of processing the reference images increase the transmission and the DPC image quality. For the investigated data, where the photon flux is naturally restricted by the laser-driven X-ray backlighter source, the DPC images retrieved with the RTA method showed higher image quality. The investigated methods of improving the CNR help extract information from reconstructed images, acquired under the harsh conditions of backlighter experiments, with a higher significance. One possible application of the presented method for evaluating the reference images could be an algorithm which iterates over every available reference image combination and uses the RTA and ATR method to find the combination with the highest SNR or lowest noise.

## Data Availability

The data presented in this article are available on request from the corresponding author.
